# Vision-related quality of life among patients with keratoconus: a cross sectional study

**DOI:** 10.3389/fmed.2023.1208911

**Published:** 2023-08-10

**Authors:** Hamzeh Al Zabadi, Mohammad Shehadeh, Lubna Amro, Nadeen Ghattass, Ibrahim Taha

**Affiliations:** ^1^Public Health Department, Faculty of Medicine and Health Sciences, An-Najah National University, Nablus, Palestine; ^2^Opthalmology Department, An-Najah National University Hospital, An-Najah National University, Nablus, Palestine; ^3^Medicine Department, Faculty of Medicine and Health Sciences, An-Najah National University, Nablus, Palestine; ^4^Optometry Department, Arab American University, Ramallah, Palestine

**Keywords:** keratoconus, quality of life, Pentacam, Palestine, vision

## Abstract

**Purpose:**

The present study aimed to evaluate the quality of life of keratoconus patients residing in Nablus city, Palestine. Furthermore, we investigated the possible correlation between Pentacam topographic indices and the quality of life of patients with keratoconus.

**Methods:**

This cross-sectional study evaluated keratoconus patients at An-Najah University Hospital in 2019, diagnosed through clinical examination and corneal tomography. A control group was randomly selected from non-keratoconus patients with normal tomography. The NEI-VFQ-25 questionnaire was administered during face-to-face interviews to compare both groups. NEI-VFQ-25 assesses vision-related quality of life and is a validated tool.

**Results:**

Keratoconus patients’ quality of life is significantly impacted, mainly in near and distant vision, general vision, mental health, and social health, but not in general health, ocular pain, color vision, role difficulties, or dependency. Visual acuity is significantly affected in both eyes of keratoconus patients.

**Conclusion:**

Patients with keratoconus exhibit a decreased quality of life related to vision, with physical, emotional, and social impairments demonstrated by the NEI-VFQ-25 when compared to controls. Since keratoconus patients are typically young adults in their productive years, understanding their concerns about their future is an important public health aspect that can aid in modifying their treatments. By addressing the specific needs of this patient population, healthcare providers can better support their long-term well-being and quality of life.

## Introduction

Keratoconus (KC) is a progressive eye disease characterized by thinning and protrusion of the cornea into a cone shape, causing irregular astigmatism and vision impairment (1). Symptoms usually present in puberty to early adulthood, with gradual progression until the fourth decade of life (2). As the disease progresses, higher-order aberrations cause increased blurring and visual distortions, interfering with daily activities such as recognizing faces, reading small print, driving, and watching TV, and can also cause difficulties in bright environments due to glare (3). KC is classified into mild, moderate, and advanced stages, with symptoms minimal in the early stages and worsening in moderate KC (4). Diagnosis is mainly made using keratometry and corneal tomography (5).

Several studies have investigated the impact of KC on patients’ vision-related quality of life. One such study by Aydin Kurna et al. (6) found that patients with KC reported lower scores on various subscales of quality of life compared to control groups, particularly in areas such as general vision, ocular pain, near vision, vision-specific mental health, vision-specific role difficulties, low contrast visual acuity, and peripheral vision (7).

Similarly, a study from Iran (8) evaluated 111 participants with KC and found that patients with higher disease duration and severity had significantly lower scores on all subscales of the National Eye Institute Visual Function Questionnaire-25 (NEI-VFQ), including mental health (8).

Results from the Collaborative Longitudinal Evaluation of Keratoconus (CLEK) study, which followed 1,166 patients over 7 years, showed that KC had a negative impact on patients’ vision-related quality of life on all scales except for ocular pain and mental health (9).

Finally, a study conducted in France involving 550 KC patients found that disease severity, a history of surgery, and a steep keratometry reading higher than 52.0 diopters were associated with a more negative impact on quality of life overall scores (10).

On the other hand, Vernal Keratoconjunctivitis (VKC) significantly affects the quality of life of individuals experiencing it. Symptoms like itching, discomfort, redness, blurred vision, and sensitivity to light can be distressing and hinder daily activities. The chronic nature of VKC, with recurring episodes, can lead to frustration and emotional distress. Additionally, the visible signs of redness, swelling, and discharge may cause embarrassment, impacting confidence and social interactions. Questionnaires and scales designed for ocular conditions, such as the Ocular Surface Disease Index (OSDI) and Impact of Vision Impairment (IVI) questionnaire, are useful tools to evaluate the functional limitations, emotional well-being, and social interactions affected by VKC (11).

As KC can greatly affect the daily activities of the patients including reading, recognizing faces, driving, watching TV, and engaging in other daily tasks that require clear vision and visual acuity, this study aims to evaluate the impact of KC on the quality of life of patients in Nablus city, Palestine, as compared to a healthy control group regularly attending follow-up appointments at An-Najah National University Teaching Hospital. By comparing these findings with regional and international data, the study seeks to underscore the significance of treating KC and improving awareness, guidance, and support to alleviate patients’ suffering. Given that KC can severely disrupt patients’ daily activities, this study’s results will be crucial in highlighting the importance of managing this disorder effectively.

## Materials and methods

### Study design, setting, and population

A comparative cross-sectional study was conducted at the ophthalmology department of An-Najah National University Teaching Hospital. Participants above 18 years old were divided into two groups: patients with KC and healthy patients who had normal eye examinations and/or tests as a comparative group. All patients with a definitive diagnosis of KC who were being routinely followed at the ophthalmology clinic at An-Najah National University Teaching Hospital were enrolled. Those with ophthalmic surgery and corneal pathologies other than KC were excluded. The control group consisted of individuals who underwent comprehensive eye exams, which confirmed their normal eye health. They had no history of keratoconus or any other corneal abnormalities. Individuals with severe eye conditions or chronic systemic diseases that could affect their quality of life or cognition were excluded. Only individuals aged 18 years or older were included in the control group.

### Sample size and sampling technique

The prevalence of KC in Palestine was estimated to be around 1.5% (12). Based on this figure and the study design, as well as to achieve a power of 80 and 95% confidence interval with a 5% acceptable error, the sample size was calculated to be around 23 participants in each group. To compensate for a possible decrease in the response rate, approximately 10% was added to the sample size, and 25 participants were enrolled in each group. The equation used to calculate the sample size was N = Z^2^P(1-P)/d^2^, where z is the z-score for 95% confidence interval, P is the estimated prevalence as a proportion, and d is the acceptable error.

A probability simple random systematic sampling technique was used to recruit subjects for the study. The exact procedure was to recruit the first keratoconous patient, but not the second; then to recruit the third keratoconous patient, but not the fourth, and so on. The same technique was applied for the healthy group recruitment until the required sample size was achieved.

### Data collection procedure and questionnaire

During the study, participants underwent comprehensive eye examinations to assess the study independent variables by Dr. Mohammad Shehadi. Those who met the inclusion criteria from both groups were enrolled. After obtaining topography findings for the 25 patients diagnosed with KC, severity levels were assessed based on Belin-ABCD criteria (13). It is worth mentioning that the same criteria of Belin–ABCD had been adapted and used on the Palestinian population previously by Shehadeh et al. (12). These criteria incorporate various topographic parameters, including corneal steepness, astigmatism, and thinnest corneal thickness, which are widely accepted indicators for keratoconus classification. Additionally, the National Eye Institute Visual Function Questionnaire-25 (NEI-VFQ-25), a previously standardized and validated quality of life questionnaire, was administered by two newly graduated MD medical doctors who previously trained on a standard delivery of the questionnaire through face-to-face interviews with the participants. The NEI-VFQ-25 is designed to assess vision-related quality of life (V-QoL) for a wide range of ocular conditions, including diabetic retinopathy, glaucoma, and macular degeneration, and is available online.

The NEI-VFQ-25 questionnaire comprises 12 subscales, including general health (2 items), general vision (2 items), ocular pain (2 items), near vision (6 items), distance vision (6 items), vision-specific social functioning (3 items), vision-specific mental health (5 items), vision-specific role difficulties (4 items), vision-specific dependency (4 items), driving (3 items), color vision (1 item), and peripheral vision (1 item). The subscale responses were graded on a scale of 0 to 100 based on published literature, where higher scores represent better VRQoL. The subscale items were averaged to form subscales, and the sum of averages resulted in a composite score (14, 15).

### Ethical and administrative procedures

The study was conducted with the approval of the An-Najah National University Institutional Review Board (IRB) and the scientific research committee at the Faculty of Medicine and Health Sciences. Prior to collecting any data, permission was obtained from the An-Najah National University administration and the CEO of the An-Najah National University Teaching Hospital. Participation was voluntary, and those who agreed to participate were asked to sign a written consent. An explanatory letter, which included the aim, importance, confidentiality, and anonymity of the information obtained, was attached to each questionnaire. The interviewer did not interfere with the participants’ choice of answer.

### Statistical analysis

After the information was collected, the data was analyzed using SPSS version 16 (SPSS Inc., Chicago, Illinois, United States) statistical software. First, the data was described using descriptive statistical methods, which included central and distribution indexes and frequency distribution. Afterwards, the Chi-square test and independent-samples t-test were used to compare qualitative and quantitative variables, respectively. In all calculations, a value of p of <0.05 was considered significant.

## Results

The study included a total of fifty patients (25 KC patients), whose demographic characteristics such as gender, age, education, and use of eye aids (contact lenses and spectacles) are presented in Table 1. The analysis revealed no significant differences in these characteristics between the groups, as indicated by a *p* value > than 0.05.

**Table 1:** Demographic characteristics of the patients [age, gender, education, eye aids use, and best corrected visual acuity (BCVA) for better and worse eye] according to the groups.

	Keratoconus group Mean ± SD or Number (%)	Control group Mean ± SD or Number (%)	*p* value
Age	26.8 ± 6.5	24.4 ± 3.7	0.115
Gender			0.172
Female	12 (63.2%)	7 (36.8%)	
Male	12 (40.0%)	18 (60.0%)	
Educational level:			0.068
High school student	6 (100%)	0 (0%)	
College student	5 (50%)	5 (50%)	
Bachelor’s degree	13 (41.9%)	18 (58.1%)	
Master’s degree	1 (33.3%)	2 (66.7%)	
Eye aids:			0.110
None	10 (47.6%)	11 (52.4%)	
Contact lens	4 (100%)	0 (0%)	
Spectacles	11 (44%)	14 (56%)	
BCVA/better eye	0.81 ± 0.28	0.99 ± 0.03	**0.003**
BCVA/worse eye	0.67 ± 0.28	0.96 ± 0.1	**0.00**

The *p*-value were significant for these values.

Out of the total of 25 patients diagnosed with KC, 18 individuals had bilateral KC while only 7 had unilateral KC. On the other hand, a total of ten cases did not utilize glasses or contact lenses. Their visual acuity ranged from 0.2 to 0.7.

In KC corneal thinning and steepening were observed in the majority of patients, with 22 individuals exhibiting this characteristic conical shape associated with KC. Furthermore, irregular astigmatism, causing blurred and distorted vision, was present in 23 patients. High myopia was seen in 18 patients, corneal scarring, a manifestation of advanced keratoconus, was observed in 9 patients contributing to further visual impairment. Based on the topography findings, the 25 patients diagnosed with keratoconus were categorized into different groups based on the severity of their condition. Among these patients, 10 were classified as having mild keratoconus, 8 were categorized as moderate cases, and 7 were identified as severe cases, according to the established classification criteria used by Belin – ABCD (13). These classifications were determined by analyzing various topographic parameters, such as corneal steepness, astigmatism, and thinnest corneal thickness, in accordance with widely accepted guidelines for keratoconus classification.

However, the results showed that the KC group had a significantly worse best-corrected visual acuity (BCVA) mean in both the better eye (*p*-value = 0.03) and worse eye (*p*-value = 0.00) compared to the control group. All subscales of NEI-VFQ-39 were lower in the KC group compared to control (Table 2).

**Table 2:** NEI-VFQ-39 subscale scores according to the groups (*p* < 0.05).

NEI-VFQ-39 scales	Keratoconus group	Control group	*p* value
General health	85.0 ± 12.5	90.0 ± 19.1	0.059
General vision	69.6 ± 16.5	90.0 ± 19.1	**0.004**
Ocular pain	66.5 ± 22.2	73.5 ± 17.8	0.29
Near vision	77.3 ± 14.4	96.5 ± 6.6	**0.001**
Distance vision	72.3 ± 18.2	86.7 ± 14.6	**0.003**
Social functioning	80.7 ± 20.8	91.0 ± 12.5	**0.04**
Mental health	56.4 ± 22.6	74.6 ± 25.5	**0.006**
Role difficulties	58.5 ± 30.3	72.5 ± 32.1	0.066
Dependency	80.0 ± 22.0	83.0 ± 28.5	0.16
Color vision	96.0 ± 11.8	100.0 ± 0.0	0.077
Peripheral vision	92.0 ± 13.9	97.0 ± 15.0	**0.03**
Overall composite score	75.84 ± 18.6	86.8 ± 17.3	0.0

The *p*-value were significant for these values.

The results of the study revealed significant differences among the subscales of vision, with near vision exhibiting the most substantial discrepancy (*p* value = 0.001), followed by distance vision (*p* value = 0.003), general vision (*p* value = 0.004), vision-specific mental health (*p* value = 0.006), peripheral vision (*p* value = 0.03), and social function (*p* value = 0.04). Further analysis showed that no statically differences were observed in quality of life among KC severity subgroups.

Then, patients diagnosed with KC were categorized into two groups based on their total score on the National Eye Institute Visual Function Questionnaire (NEI-VFQ-25). The first group comprised of patients who had a good quality of life, with a total score of all subscales greater than 75, while the second group consisted of patients who had a poor quality of life, with a total score of less than 75. It is worth noting that the mean score for both groups was 75.

To assess the relationship between the NEI-VFQ-25 scores and Pentacam topography indices, we compared the indices between the two groups. The analysis of the data indicated that there was no significant correlation between the two groups for all the Pentacam topography indices studied. The detailed results are presented in Table 3.

**Table 3:** Pentacam tomographic indices.

	Good QOL Mean ± SD	Poor QOL Mean ± SD	*p* value
ISV OD	72.6 ± 50.0	71.7 ± 33.8	0.81
ISV OS	85.2 ± 37.0	72.3 ± 39.26	0.56
IVA OD	0.661 ± 0.41	0.664 ± 0.44	0.53
IVA OS	0.765 ± 0.42	0.740 ± 0.46	0.84
KI OD	1.20 ± 0.16	1.17 ± 0.10	0.41
KI OS	1.19 ± 0.16	1.14 ± 0.13	0.58
CKI OD	1.05 ± 0.08	1.05 ± 0.04	0.60
CKI OS	1.03 ± 0.07	1.03 ± 0.06	0.66
IHA OD	31.6 ± 23.6	29.1 ± 17.7	0.42
IHA OS	28.8 ± 23.3	32.53 ± 22.14	0.97
IHD OD	0.09 ± 0.09	0.09 ± 0.06	0.98
IHD OS	0.10 ± 0.66	0.10 ± 0.67	0.83
Rmin OD	6.3 ± 0.97	6.2 ± 0.72	0.93
Rmin OS	6.1 ± 0.83	6.4 ± 0.74	0.47
Astigmatism OD	3.35 ± 1.58	3.25 ± 2.11	0.10
Astigmatism OS	3.8 ± 2.87	2.5 ± 1.41	0.09
K1 OD	47.43 ± 5.82	46.08 ± 5.42	0.73
K1 OS	47.54 ± 6.51	45.16 ± 4.71	0.55
K2 OD	50.79 ± 4.05	50.09 ± 4.63	0.75
K2 OS	51.4 ± 7.82	47.68 ± 4.49	0.17
Kmax OD	55.1 ± 10.95	54.65 ± 6.53	0.68
Kmax OS	56.37 ± 7.85	52.95 ± 6.89	0.45
RMS OD	8.65 ± 8.31	9.05 ± 5.3	0.83
RMS OS	10.32 ± 4.94	9.34 ± 6.02	0.35
Front Q value OD	−0.81 ± 0.68	−0.78 ± 0.42	0.47
Front Q value OS	−0.75 ± 0.74	−0.62 ± 0.43	0.20
Back Q value OD	−0.96 ± 0.60	−1.01 ± 0.50	0.85
Back Q value OS	−0.90 ± 0.70	−0.84 ± 0.50	0.35
Anterior elevation OD	18.2 ± 20.1	16.7 ± 10.3	0.49
Anterior elevation OS	18.1 ± 16.9	10.5 ± 16.3	0.96
Posterior elevation OD	53.3 ± 33.3	52.4 ± 23.5	0.75
Posterior elevation OS	61.9 ± 34.6	52.7 ± 24.0	0.15
SI OD	−6.25 ± 5.42	−6.08 ± 3.86	0.60
SI OS	−4.83 ± 4.63	−4.80 ± 4.21	0.62

SI, superior minus inferior thickness; CKI, central keratoconus index; IHA, index of height asymmetry; IHD, index of height decentration; ISV, index of surface variance; IVA, index of vertical asymmetry; KI, keratoconus index; Max, maximum; Rmin, minimum sagittal curvature; RMS, room mean squares.

## Discussion

KC is a challenging and complex eye disease that typically manifests during adolescence. This progressive condition is characterized by the steepening and thinning of the cornea, resulting in a cone-like shape that affects the patient’s vision (1, 16, 17). Although the underlying cause of KC is not yet fully understood, it is widely believed to be a result of the interplay between genetic and environmental factors (16).

While KC may initially be asymptomatic, it can significantly impact a patient’s visual acuity and quality of life in the later stages of the disease (18). Diagnosis of KC is typically made through a comprehensive eye exam, which includes a thorough evaluation of the cornea’s topography and thickness (19). However, KC patients eventually present with visual impairment in the later stages, along with bio-microscopic signs of KC including marked stromal thinning, Fleischer’s ring, Vogt’s striae and corneal apical scarring (20).

Early intervention and management are crucial in slowing the progression of KC and preserving vision (21). Treatment options include corrective lenses, corneal cross-linking, and in advanced cases, corneal transplantation. Patients with KC require regular monitoring and follow-up care to ensure optimal outcomes (22).

Ongoing research is essential to gain a better understanding of the underlying mechanisms of KC and to develop more effective treatments. Additionally, public awareness campaigns and education initiatives can help improve early detection and diagnosis, leading to better outcomes for patients with KC (23).

Several previous reports have documented the impact of KC on NEI-VFQ-25 scales (24, 25). However, there is limited research on the impact of KC on vision-related quality of life (VR-QoL) compared to normal individuals. Therefore, we aimed to compare the mean NEI-VFQ scores of 50 age-and sex-matched subjects who had referred to the cornea clinic due to other ocular diseases, except KC.

To address this gap, we utilized the NEI-VFQ-25, a vision-targeted questionnaire that assesses the impact of visual problems on various aspects of an individual’s life, including physical, emotional, and social functioning.

Our sample consisted of 50 patients, of which 25 had been previously diagnosed with KC, while the other 25 had other visual problems. We found no significant differences in the NEI-VFQ-25 subscale item scores when analyzing age (*p*-value = 0.115), gender (*p*-value = 0.172), education (*p* value = 0.068), or use of eye aids (*p*-value = 0.11). However, we did observe significant differences in visual acuity, with both the better and worse eyes showing significantly better visual acuity in the control group compared to the KC group (*p* value = 0.003 and *p* value = 0.00, respectively, as shown in Table 1).

The NEI-VFQ-25 subscales include general health, general vision, ocular pain, near vision, distance vision, social functioning, mental health, role difficulties, dependency, color vision, and peripheral vision. However, in the KC group, all subscales had lower scores compared to the control group. Specifically, the overall composite score for the KC group was 75.84 ± 18.6, which was significantly lower than the overall composite score of the control group, which was 86.8 ± 17.3 (Table 2), highlighting the impact of KC on vision-related quality of life.

The differences between the KC and control groups were particularly pronounced in the subscales of near vision (*p* value = 0.001), distance vision (*p* value = 0.003), general vision (*p* value = 0.004), vision-specific mental health (*p* value = 0.006), peripheral vision (*p* value = 0.03), and social function (*p* value = 0.04). However, the differences were less significant in the subscales of general health (*p* value = 0.059), ocular pain (*p* value = 0.29), color vision (*p* value = 0.07), role difficulties (*p* value = 0.066), and dependency (*p* value = 0.16).

Furthermore, the KC patients were divided into two groups based on their total score on the NEI-VFQ-25 questionnaire. The first group consisted of 13 patients with a score above 75, indicating good quality of life (QOL), while the second group consisted of 12 patients with a score below 75, indicating poor QOL.

In addition to the questionnaire results, several Pentacam indices were measured for all patients, including ISV, IVA, KI, CKI, IHA, IHD, Rmin, RMS, K1, K2, Kmax, astigmatism, front Q value, back Q value, anterior elevation, posterior elevation, for both the right (OD) and left (OS) eyes.

After the division of the KC group into two subgroups based on their QOL score, the Pentacam indices were analyzed for both groups to determine if there was any correlation between the indices and quality of life. However, the analysis did not reveal any significant difference between the Pentacam indices and quality of life (*p* value >0.05), as shown in Table 3.

The results of these studies show that the NEI-VFQ-25 has acceptable discriminant validity, as patients with KC tend to have lower scores on this measure than individuals without ocular disease. This suggests that the NEI-VFQ-25 is an effective tool for distinguishing between different groups based on their visual function and quality of life.

Furthermore, the NEI-VFQ-25 has demonstrated good internal consistency in these studies, indicating that the items on the measure are measuring the same construct and are thus reliable. This means that the NEI-VFQ-25 can be used in different types of ocular disease and across different age groups, providing a consistent and reliable measure of visual function and quality of life.

The findings of this study confirm previous research regarding the social burden of KC, including its impact on near vision, driving, daily work, and mental health related to vision and social functioning (7). While KC patients may not generally experience low overall health, this is likely due to the fact that most patients are in the adult age group and do not have other chronic diseases that are more common among the elderly.

Interestingly, our study highlights the results of previous research that suggest that ocular pain and color vision subscales are less affected in KC patients than other subscales (6). These findings may provide valuable insights for healthcare providers working with KC patients, as they can help guide treatment plans and improve overall patient outcomes.

To the best of our knowledge, this is the first study to report on the Quality of Life (QOL) of KC patients in Palestine using a validated tool. However, our study has several limitations that should be taken into account. Firstly, our sample size was small, which may have contributed to the lack of significance in the pentacam indices. A larger sample size may have resulted in more significant findings. Secondly, our sample may not be representative of all Palestinian KC patients, as it was drawn solely from a single clinic in Nablus and may not reflect the experiences of patients from other regions.

### Study limitations

As part of our baseline study, we included questions in the questionnaire regarding the utilization of vision aids. One of the primary objectives of our study was to evaluate the impact of vision-related quality of life, employing the National Eye Institute Visual Function Questionnaire (NEI-VFQ-25). However, it is crucial to acknowledge that our ability to conduct a detailed sub-group analysis was limited due to the small size of our sample. While our study did not specifically focus on exploring differences related to vision aids, we recognize the significance of investigating this aspect within the context of KC. Hence, we highly recommend. we further future studies with larger sample size.

Another limitation of our study is the restricted geographic scope, as it only pertains to the Palestinian population. This may limit the generalizability of our findings to other populations with potentially different demographic, genetic, and environmental characteristics. Therefore, caution should be exercised when extrapolating our results to broader populations.

## Conclusion

In conclusion, our study indicates that KC patients experience a significant reduction in vision-related quality of life, evidenced by physical, emotional, and social impairments as assessed by the NEI-VFQ-25 compared to the control group. Given that KC patients are typically young adults in their productive years, it is crucial to recognize their concerns about their future as an essential public health consideration that can inform treatment modifications. Our findings also underscore the validity, reliability, and applicability of the NEI-VFQ-25 in further investigations. Overall, these results highlight the significant impact of KC on patients’ daily lives and emphasize the need for tailored interventions to enhance their quality of life.

## Data availability statement

The original contributions presented in the study are included in the article/supplementary materials, further inquiries can be directed to the corresponding author.

## Ethics statement

The studies involving human participants were reviewed and approved by An-Najah National University IRB committee. The patients/participants provided their written informed consent to participate in this study.

## Author contributions

HZ, MS, LA, NG, and IT contributed to the study conception and design and commented on previous versions of the manuscript. Material preparation, data collection and analysis were performed by HZ, MS, LA, and NG. The draft of the manuscript was written by HZ, IT, LA, and NG. All authors contributed to the article and approved the submitted version.

## Conflict of interest

The authors declare that the research was conducted in the absence of any commercial or financial relationships that could be construed as a potential conflict of interest.

## Publisher’s note

All claims expressed in this article are solely those of the authors and do not necessarily represent those of their affiliated organizations, or those of the publisher, the editors and the reviewers. Any product that may be evaluated in this article, or claim that may be made by its manufacturer, is not guaranteed or endorsed by the publisher.

## Abbreviations

BCVA: best corrected visual acuity
CLEK: collaborative longitudinal evaluation of keratoconus
SI: superior minus inferior thickness
CKI: central keratoconus index
IHA: index of height asymmetry
IHD: index of height decentration
ISV: index of surface variance
IVA: index of vertical asymmetry
KI: keratoconus index
Max: maximum
Rmin: minimum sagittal curvature
RMS: room mean squares
Qol: quality of life
